# Evaluation of macrophage activation syndrome in hospitalised patients with Kikuchi-Fujimoto disease based on the 2016 EULAR/ACR/PRINTO classification criteria

**DOI:** 10.1371/journal.pone.0219970

**Published:** 2019-07-18

**Authors:** Sung Soo Ahn, Byeori Lee, Dam Kim, Seung Min Jung, Sang-Won Lee, Min-Chan Park, Yong-Beom Park, Yong Gil Hwang, Jason Jungsik Song

**Affiliations:** 1 Division of Rheumatology, Department of Internal Medicine, Yonsei University College of Medicine, Seoul, South Korea; 2 Department of Internal Medicine, Albert Einstein Medical Center Philadelphia, Philadelphia, Pennsylvania, United States of America; 3 Department of Medicine, Division of Rheumatology and Clinical Immunology, University of Pittsburgh, Pittsburgh, Pennsylvania, United States of America; 4 Institute for Immunology and Immunological Diseases, Yonsei University College of Medicine, Seoul, South Korea; Soroka University Medical Center, ISRAEL

## Abstract

**Background:**

To evaluate the impact of macrophage activation syndrome (MAS) on clinical features in patients with Kikuchi-Fujimoto disease (KFD) and to compare the features of MAS in KFD with those of adult-onset Still’s disease (AOSD) and systemic lupus erythematosus (SLE).

**Methods:**

The medical records of febrile patients hospitalised with KFD between November 2005 and April 2017 were reviewed. Patients fulfilling the 2016 classification criteria for MAS were classified as having MAS. Clinical and laboratory features of patients with KFD with and without MAS were evaluated. Poor hospitalisation outcomes were defined as intensive care unit admission or in-hospital mortality. The treatment outcomes of MAS in KFD, AOSD, and SLE were also compared.

**Results:**

Among 78 patients hospitalised with KFD, 24 (30.8%) patients had MAS during admission. Patients with KFD and MAS more frequently required glucocorticoid treatment (66.7% vs 40.7%, p = 0.036) and had longer hospital stays than patients with KFD without MAS (12.5 vs 8.5 days, p<0.001). In addition, patients with MAS had worse hospitalisation outcomes than patients without MAS (29.2% vs. 0.0%, p<0.001). Among patients with MAS in KFD, AOSD, and SLE, the number of patients requiring glucocorticoid treatment after 3 months was significantly lower among patients with MAS and KFD (KFD 33.3%, AOSD 88.9%, SLE 100%, p<0.001).

**Conclusions:**

The presence of MAS in KFD was associated with adverse clinical outcomes including higher steroid usage and worse hospitalisation outcomes. However, compared to those with AOSD and SLE, patients with MAS and KFD were less likely to require long-term glucocorticoid treatment.

## Introduction

Kikuchi-Fujimoto disease (KFD), also known as histiocytic necrotising lymphadenitis, is a rare idiopathic inflammatory disease first described in 1972 by Kikuchi and Fujimoto et al [1, 2]. KFD mainly affects adults younger than 40 years of age and is predominant among Asian populations [3]. The aetiology of KFD is still unknown although various inciting agents, including the Epstein-Barr virus (EBV), human herpesvirus types 6, 7, and 8, herpes simplex virus, and HIV, have been suggested as possible causative agents [1, 2, 4]. KFD typically presents as persistent cervical lymphadenopathy with or without fever [5]. Some patients manifest non-specific systemic symptoms including night sweats, weight loss, nausea, vomiting, hepatosplenomegaly, and headache [6, 7]. The clinical characteristics of KFD can resemble those of malignant lymphoma, metastatic carcinoma, infectious lymphadenitis, systemic autoimmune diseases, and *Mycobacterium tuberculosis* infections [8–10]. The overall prognosis of KFD is favourable with a benign, self-limiting course. Although there is no specific treatment for KFD, supportive management with analgesics and antipyretics is usually adequate, leading to spontaneous resolution in several months [6].

In KFD cases with severe clinical presentations, few case reports described co-existence of hemophagocytic lymphohistiocytosis (HLH) [11–18]. HLH is a syndrome of excessive proinflammatory cytokine production due to uncontrolled expansion of T lymphocytes and macrophages, which is characterised by hemophagocytosis, intractable fever, hepatosplenomegaly, cytopenia, hypertriglyceridemia, and hypofibrinogenemia [19, 20]. Although primary HLH is associated with genetic defects in the perforin-mediated cytolytic pathway, macrophage activation syndrome (MAS) is considered to be a secondary form of HLH associated with malignancy, infection, and autoimmune diseases [21]. Because viral infections such as EBV are known to cause HLH by triggering uncontrolled lymphoproliferation [22], there is a concern that KFD can be associated with MAS. While MAS is a potentially life-threatening condition that requires early detection, MAS-specific diagnostic criteria have not yet been fully established. Recently, new classification criteria for MAS were developed by a collaboration of international study groups and were validated in patients with systemic juvenile idiopathic arthritis (sJIA) with excellent specificity [23, 24]. We previously demonstrated that these criteria were also useful for the identification of patients with MAS in adult-onset Still’s disease (AOSD) and systemic lupus erythematosus (SLE) to predict poor hospitalisation outcomes [25, 26].

Although MAS may follow a severe and fatal course, the incidence and clinical characteristics of MAS-complicated KFD are still not well understood. In our study, we aimed to evaluate the impact of MAS on the clinical features of KFD and to compare the clinical and laboratory features of patients with MAS complicating KFD, AOSD, and SLE.

## Material and methods

### Patient selection and study design

We retrospectively reviewed the clinical and laboratory data of patients with a diagnosis of KFD between November 2005 and April 2017 in two tertiary hospitals in the Yonsei University Health System located in Shinchon and Gangnam district of Seoul, Korea. The inclusion criteria were as follows: (i) patients who were admitted with fever; (ii) patients with diagnosis of KFD that was confirmed pathologically; (iii) patients with ferritin level assessment; (iv) patients without concurrent autoimmune disease/infection. Finally, 78 patients were included in this study. The flowchart for patient selection is shown in S1 Fig. The clinical and laboratory data of patients with MAS in AOSD and SLE according to the 2016 classification criteria were obtained using a pre-existing dataset used in a previous study [25, 26], and all data were fully anonymized for analysis. This study was conducted in accordance with the Declaration of Helsinki and was approved by the Institutional Review Board of Severance Hospital (IRB approval number 4-2018-0624). The requirement to obtain informed consent was waived because of the retrospective nature of the study.

### Definition of patients with MAS according to the 2016 classification criteria

According to the 2016 EULAR/ACR/PRINTO classification criteria for MAS [23, 24], we classified patients as having MAS when they presented with fever, a ferritin level of ≥684 ng/mL, and fulfilled more than 2 of the following 4 criteria: platelet count ≤181,000/mL, aspartate aminotransferase (AST) level >48 units/L, triglyceride level >156 mg/dL, and fibrinogen level ≤360 mg/dL. In addition, patients with MAS were divided into two groups: patients having MAS on admission and those who developed MAS after admission.

### Collection of clinical and laboratory variables

The clinical data collected included age, sex, length of hospital stay, in-hospital mortality, intensive care unit admission, and treatment. Poor hospitalisation outcomes were defined as intensive care unit admission or in-hospital mortality. The clinical manifestations of KFD included the presence of hepatomegaly, splenomegaly, skin rash, and joint pain. Baseline laboratory variables included white blood cells, neutrophils, lymphocyte and platelet counts, erythrocyte sedimentation rate, and levels of haemoglobin, C-reactive protein (CRP), creatinine, AST, alanine aminotransferase (ALT), albumin, ferritin, lactate dehydrogenase (LDH), fibrinogen, and triglycerides. As fibrinogen and triglyceride levels were not measured regularly, the levels obtained within 3 days of the date of laboratory data selection were included in the analysis.

### Calculation of the hemophagocytic syndrome score (HScore) and patients fulfilling the HLH-2004 criteria

The HScore was developed to evaluate individual risk for the presence of hemophagocytic syndrome [27] and is also useful to identify the risk of having reactive hemophagocytic syndrome in patients with rheumatic diseases [28]. The HScore and the probability of having hemophagocytic syndrome were calculated using an online calculator (saintantoine.aphp.fr/score). Furthermore, the proportion of patients fulfilling the HLH-2004 criteria was estimated as previously described [29].

### Statistical analysis

Continuous variables were presented as medians with interquartile ranges (IQR), and categorical variables were presented as frequencies and percentages. Continuous variables were compared using the Kruskal–Wallis test or Mann–Whitney U test, and categorical variables were compared using the chi-square or Fisher’s exact test, as appropriate. Kaplan-Meier analysis and the log-rank test were used to compare the survival probability among the groups. All statistical analyses were performed using MedCalc statistical software version 18.6 (MedCalc Software, Ostend, Belgium) or GraphPad Prism software version 5.0 (GraphPad Software, San Diego, California, USA). In all statistical analyses, a two-tailed p<0.05 was considered statistically significant.

## Results

### Baseline characteristics of patients with and without MAS

**Fig 1:**
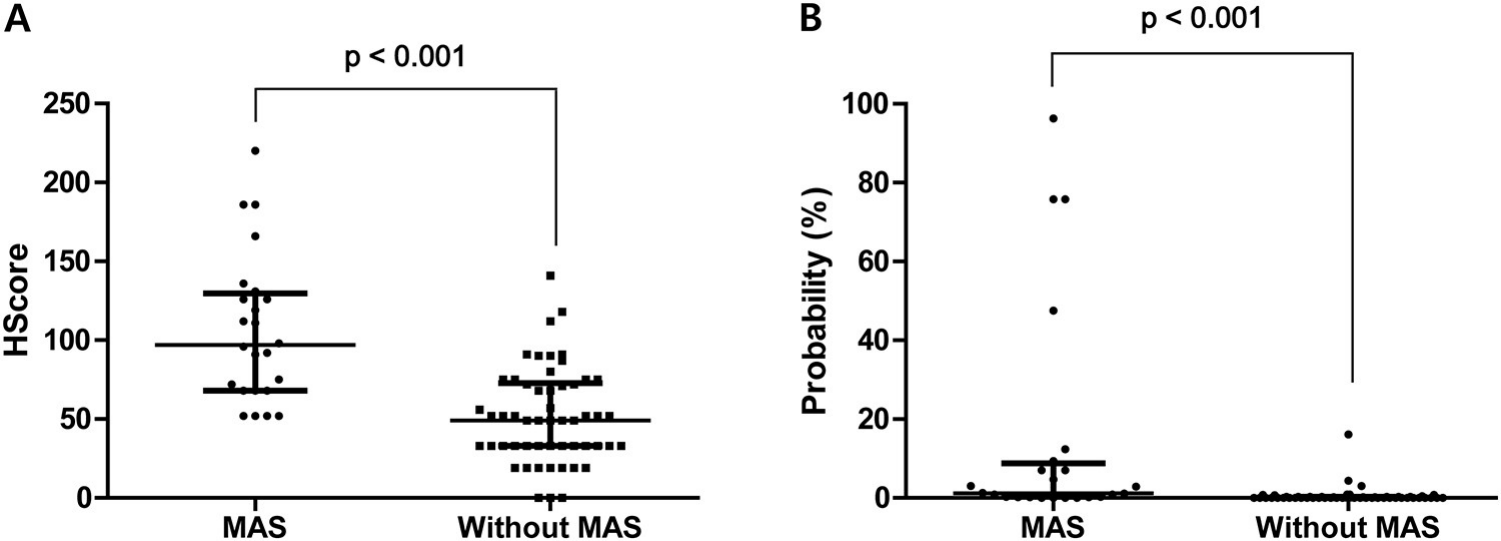
HScore and the probability of having hemophagocytic syndrome in patients with KFD with and without MAS. Comparison of HScore (A) and the probability of having hemophagocytic syndrome (B) in patients with KFD with and without MAS. HScore, hemophagocytic syndrome score; KFD, Kikuchi-Fujimoto disease; MAS, macrophage activation syndrome.

Of the 78 patients included in the study, 24 (30.8%) were classified as having MAS during admission (Table 1). Among the 24 patients, 20 (83.3%) had MAS on admission, and 4 (16.7%) developed MAS after admission. Although the clinical manifestations were not different between patients with and without MAS, patients with MAS were older and had a longer hospital stays, and the proportion of patients with poor hospitalisation outcomes was significantly higher in patients with MAS (29.2% vs. 0.0%, p<0.001). In addition, the median HScore (97.0 [IQR 68.0–128.5] vs 49.0 [IQR 33.0–72.0]) and the probability of having hemophagocytic syndrome were higher in patients with MAS than in patients without MAS (all p<0.001) (Fig 1A and 1B). Furthermore, the proportion of patients requiring glucocorticoid treatment was significantly higher in patients with MAS (66.7% vs 40.7%, p = 0.036). Regarding laboratory findings, patients with MAS had higher CRP, ALT, and LDH levels and a lower lymphocyte count and albumin levels than those without MAS. Among the laboratory variables selected for MAS classification, higher AST and ferritin levels and lower platelet counts were observed in patients with MAS; however, the differences between triglyceride and fibrinogen levels were not significant (Table 2). Of the 19/78 (24.4%) patients who had undergone a bone marrow study, only 2 patients in the MAS group were found to have hemophagocytosis.

**Table 1:** Clinical characteristics of patients with KFD with and without MAS.

	Without MAS (n = 54)	With MAS (n = 24)	p-value
**Demographic data**			
Age, years	26.0 (21.0–35.0)	40.0 (32.5–55.0)	<0.001
Female sex	37 (68.5)	15 (62.5)	0.605
**Clinical manifestations**			
Hepatomegaly	6 (11.1)	0 (0.0)	0.169
Splenomegaly	12 (22.2)	7 (29.2)	0.512
Skin rash	8 (14.9)	7 (29.2)	0.140
Joint pain	1 (1.9)	3 (12.5)	0.084
**Treatment**			
Glucocorticoid treatment	22 (40.7)	16 (66.7)	0.036
Immunosuppressive treatment	1 (1.9)	3 (12.5)	0.084
Hydroxychloroquine	1 (1.9)	2 (8.3)	
Cyclosporine	0 (0.0)	1 (4.2)	
**Clinical course**			
Length of stay in hospital (days)	8.5 (6.0–11.0)	12.5 (10.5–32.0)	<0.001
In-hospital mortality	0 (0.0)	5 (20.8)	0.002
Intensive unit care	0 (0.0)	7 (29.2)	<0.001

Data expressed as median (interquartile range) or n (%). KFD, Kikuchi-Fujimoto disease; MAS, macrophage activation syndrome.

**Table 2:** Comparison of baseline laboratory findings of patients with and without MAS.

Variable	Without MAS (n = 54)	With MAS (n = 24)	p-value
**General laboratory variables**			
WBC count (/μL)	3475.0 (2350.0–5000.0)	3285.0 (2335.0–5905.0)	0.996
Hemoglobin (g/dL)	12.5 (11.7–13.4)	11.7 (10.3–13.2)	0.102
Neutrophil count (/μL)	1925.0 (1310.0–3050.0)	2206.5 (1375.0–4833.5)	0.378
Lymphocyte count (/μL)	1045.0 (860.0–1370.0)	600.0 (490.0–940.0)	<0.001
ESR (mm/h)	48.5 (27.0–64.0)	52.5 (39.5–72.5)	0.242
CRP (mg/L)	15.0 (7.5–37.1)	52.6 (27.7–97.1)	<0.001
Creatinine (mg/dL)	0.7 (0.6–0.8)	0.7 (0.5–1.1)	0.349
ALT (IU/L)	21.5 (14.0–38.0)	44.5 (23.0–153.0)	0.003
Albumin (g/dL)	4.0 (3.7–4.3)	3.4 (3.1–3.7)	<0.001
LDH (IU/L)	439.5 (291.0–611.0)	771.5 (460.0–1213.5)	0.003
**Laboratory variables selected for MAS classification**			
Platelet count (×1000/μL)	195.5 (155.0–260.0)	139.0 (94.5–184.0)	<0.001
AST (IU/L)	29.0 (22.0–39.0)	93.5 (62.5–191.0)	<0.001
Ferritin (ng/mL)	242.8 (112.0–405.2)	1449.7 (812.9–4463.1)	<0.001
Triglyceride (mg/dL)^¶^	89.0 (60.0–106.5)	133.0 (90.3–161.3)	0.076
Fibrinogen (mg/dL)^§^	342.0 (338.0–346.0)	317.5 (227.5–401.0)	0.695
Bone marrow biopsy findings*			
Presence of hemophagocytosis	0/7 (0.0)	2/12 (16.7)	0.509

Data are expressed as median (interquartile range) or n (%). MAS, macrophage activation syndrome; WBC, white blood cell; ESR, erythrocyte sedimentation rate; CRP, C-reactive protein; ALT, alanine aminotransferase; LDH, lactate dehydrogenase; AST, aspartate aminotransferase.

^¶^Number confined to patients who underwent each test (n = 23).

^§^Number confined to patients who underwent each test (n = 10).

*Number confined to patients who underwent each test (n = 19).

### Clinical and laboratory findings of patients with KFD and in-hospital mortality

**Fig 2:**
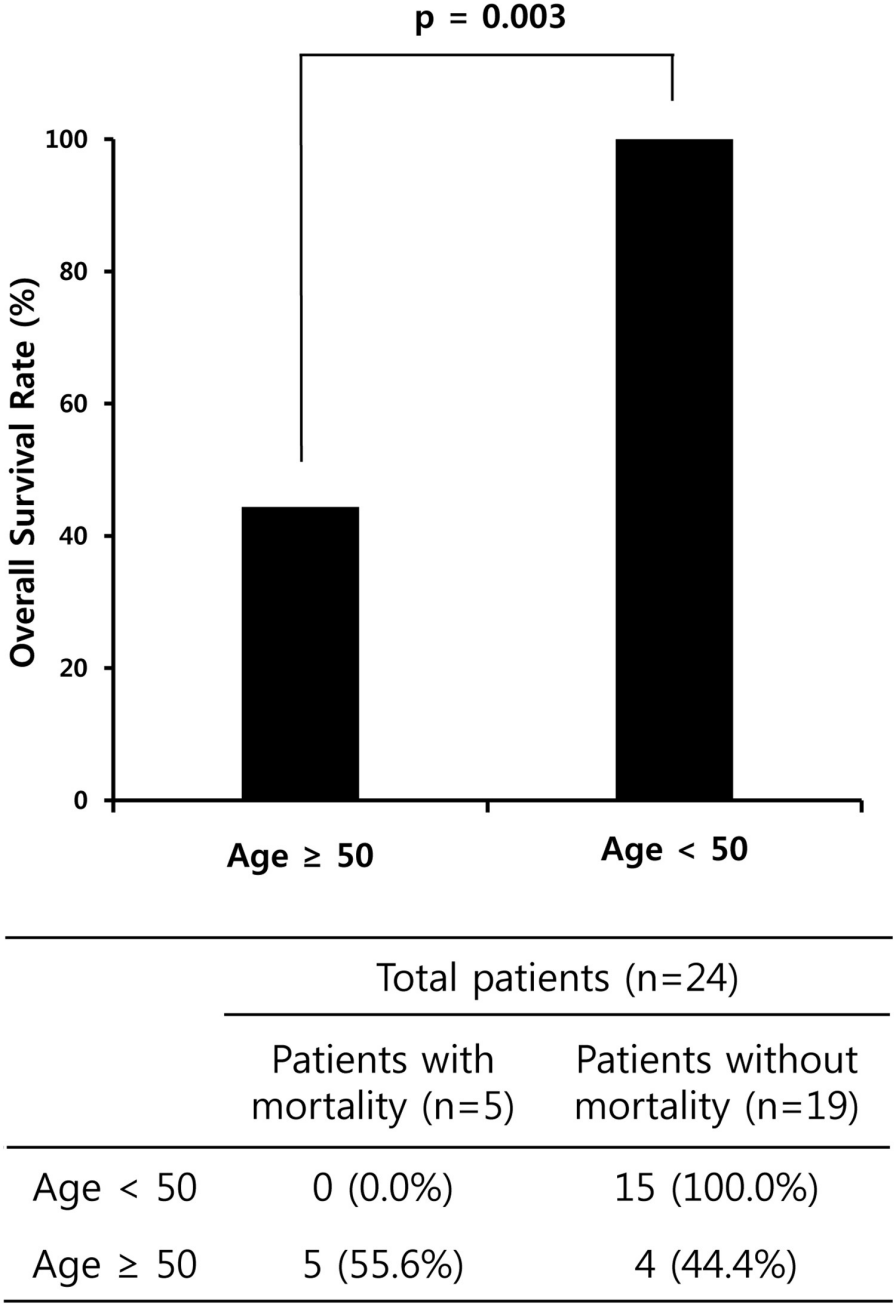
Comparison of the overall survival rate of patients with KFD and MAS according to age. In MAS patients with KFD, patients aged ≥50 years had a higher mortality rate than those aged <50 years. KFD, Kikuchi-Fujimoto disease; MAS, macrophage activation syndrome.

As MAS in KFD is not a well-described complication, we further analysed the clinical and laboratory features of patients with KFD and in-hospital mortality (Table 3). All patients in our study who died in the hospital had MAS, were older than 50 years of age and did not have any specific clinical manifestations. Among MAS patients with KFD (n = 24), patients aged ≥50 years had a higher mortality rate than those aged <50 years (p = 0.003) (Fig 2). During the disease course, patients with MAS had increasing ferritin and AST levels, while platelet counts decreased. Most deaths occurred within 90 days, and among the five patients who died, the cause of death was multi-organ failure in 4 patients and sepsis in 1 patient (Table 3). Hepatosplenomegaly was only observed in one patient, and no patients had co-existing comorbidities with the exception of gout in one patient.

**Table 3:** The clinical and laboratory features of patients with KFD and in-hospital mortality.

Case number	Age/Sex	Main symptom	Initial/highest ferritin level (ng/mL)	Initial/lowest platelet count (/μL)	Initial/highest AST level (IU/L)	Time to death (days)	Cause of death
1	68/Male	Fever	13308.3/15000.0	133000/27000	178/24650	Death in day 8	Multi-organ failure
2	52/Female	Fever, skin rash, joint pain	14399.9/14399.9	69000/20000	132/134	Death in day 11	Multi-organ failure
3	56/Male	Fever, myalgia	441.8/1896.6	346000/7000	204/8320	Death in day 24	Sepsis
4	61/Female	Fever, headache, nausea	15363.6/15363.6	92000/16000	96/394	Death in day 32	Multi-organ failure
5	57/Female	Sore throat, fever	528.9/38765.3	97000/21000	22/483	Death in day 281	Multi-organ failure

KFD, Kikuchi-Fujimoto disease.

### Clinical and laboratory features among patients with MAS in KFD, AOSD, and SLE

Next, we compared the clinical and laboratory features among patients with MAS in KFD, AOSD and SLE. Clinical and laboratory data of patients with AOSD or SLE and MAS used for comparison were obtained from the dataset of previous studies [25, 26]. Patients with AOSD were older than those with SLE, and the proportion of female patients was the lowest in patients with KFD. The presence of lymphadenopathy was significantly higher in patients with KFD than in those with AOSD or SLE (Table 4). There were no differences in the median HScore (KFD 97.0 [IQR 68.0–128.5] vs AOSD 108.5 [IQR 102.0–150.0] vs SLE 111.0 [IQR 76.0–137.0], p = 0.296) or the probability of having hemophagocytic syndrome among patients with MAS in KFD, AOSD, and SLE (S2A and S2B Fig, p = 0.296). In addition, the number of patients fulfilling the HLH-2004 criteria was 2/24 (8.3%), 6/36 (16.7%), and 3/54 (5.6%) in KFD, AOSD, and SLE, respectively, without a statistical difference (p = 0.210). Comparison of laboratory variables between the groups showed that elevated white blood cells, neutrophils, lymphocyte count, and CRP levels were prominent in patients with AOSD, while decreased haemoglobin and albumin levels were characteristic in patients with SLE. Regarding laboratory variables selected for MAS classification, platelet count and ferritin levels were the highest in patients with AOSD, while triglyceride levels were the lowest in patients with KFD (Table 4).

**Table 4:** Clinical and laboratory features in patients with MAS in KFD, AOSD, and SLE.

Variable	KFD (n = 24)	AOSD (n = 36)	SLE (n = 54)	p-value
**Demographic data**				
Age, years	40.0 (32.5–55.0)	47.5 (36.0–57.5)**	37.0 (26.0–49.0)	0.010
Female sex	15 (62.5)^b^	34 (94.4)	46 (85.2)^§^	0.004
**Clinical manifestations**				
Hepatomegaly	0 (0.0)	4 (11.1)	8 (14.8)	0.143
Splenomegaly	7 (29.2)	10 (27.8)	16 (29.6)	0.982
Lymphadenopathy	24 (100.0)^c^	16 (44.4)	29 (53.7)^§§§^	<0.001
**General laboratory variables**				
WBC count (/μL)	3285.0 (2335.0–5905.0)^c^	10630.0 (6915.0–6130.0)***	2830.0 (1800.0–6210.0)	<0.001
Hemoglobin (g/dL)	11.7 (10.3–13.2)	10.9 (10.0–11.7)**	10.1 (8.7–11.0)^§§§^	<0.001
Neutrophil count (/μL)	2206.5 (1375.0–4833.5)^c^	8575.0 (4780.0–14325.0)***	2015.0 (1230.0–4320.0)	<0.001
Lymphocyte count (/μL)	600.0 (490.0–940.0)^a^	899.0 (655.0–1225.0)***	491.4 (310.0–790.0)	<0.001
ESR (mm/h)	52.5 (39.5–72.5)	61.0 (29.0–83.0)	42.5 (29.0–70.0)	0.186
CRP (mg/L)	52.6 (27.7–97.1)^a^	96.4 (50.2–143.8)***	13.5 (5.5–59.9)^§§^	<0.001
ALT (IU/L)	44.5 (23.0–153.0)	36.0 (23.5–117.5)	38.5 (24.0–68.0)	0.729
Albumin (g/dL)	3.4 (3.1–3.7)	3.1 (3.0–3.6)*	2.9 (2.4–3.3)^§§^	0.011
**Laboratory variables selected for MAS classification**				
Platelet count (×1000/μL)	139.0 (94.5–184.0)^a^	203.5 (140.0–292.0)***	109.5 (91.0–155.0)	<0.001
AST (IU/L)	93.5 (62.5–191.0)	76.5 (42.0–207.5)	89.0 (50.0–183.0)	0.778
Ferritin (ng/mL)	1449.7 (812.9–4463.1)^b^	6773.4 (2567.1–14610.2)***	1483.5 (754.9–2324.7)	<0.001
Triglyceride (mg/dL)	133.0 (90.3–161.3)	168.0 (126.7–223.5)	173.0 (145.5–274.0)^§§^	0.026
Fibrinogen (mg/dL)	317.5 (227.5–401.0)	302.0 (202.5–406.3)	253.0 (170.0–329.0)	0.320
**Clinical course and treatment outcome**				
Length of stay in hospital (days)	12.5 (10.5–32.0)	22.5 (13.0–41.5)	19.0 (14.0–29.0)	0.257
Glucocorticoid treatment cessation at 3 months^†^	16 (66.7)^c^	4 (11.1)*	0 (0.0)^§§§^	<0.001
Number of Immunosuppressive treatments administered^‡^				0.018
0–1	24 (100.0)^b^	26 (72.2)	45 (83.3)^§^	
≥2	0 (0.0)	10 (27.8)	9 (16.7)	

Data expressed as median (interquartile range) or n (%).

^†^Glucocorticoid treatment and the dosage of glucocorticoids in patients with mortality or who were lost to follow-up were counted on the last date of follow-up.

^‡^Includes treatment with disease modifying anti-rheumatic drugs, biologics, cyclosporine, and etoposide/cyclophosphamide.

^a^Difference between patients with KFD and AOSD. p < 0.05;

^b^Difference between patients with KFD and AOSD, p < 0.01;

^c^Difference between patients with KFD and AOSD, p < 0.001.

^§^Difference between patients with KFD and SLE. p < 0.05;

^§§^Difference between patients with KFD and SLE, p < 0.01;

^§§§^Difference between patients with KFD and SLE, p < 0.001.

*Difference between patients with AOSD and SLE, p < 0.05;

**Difference between patients with AOSD and SLE, p < 0.01;

***Difference between patients with AOSD and SLE, p < 0.001.

MAS, macrophage activation syndrome; KFD, Kikuchi-Fujimoto disease; AOSD, adult onset Still’s disease; SLE, systemic lupus erythematosus; WBC, white blood cell; ESR, erythrocyte sedimentation rate; CRP, C-reactive protein; ALT, alanine aminotransferase; AST, aspartate aminotransferase.

### Comparison of 90-day mortality and treatment among patients with MAS in KFD, AOSD, and SLE

To evaluate the prognosis of patients with MAS in different diseases, we compared the 90-day mortality among patients with MAS in KFD, AOSD, and SLE by using Kaplan-Meier analysis with the log-rank test. The number of patients with 90-day mortality in KFD, AOSD, and SLE was 4 (16.7%), 12 (33.3%), and 15 (27.8%) patients, respectively. Kaplan-Meier survival analysis showed that the survival probability among patients with MAS in KFD, AOSD, and SLE was not statistically significant (p = 0.427) (S3 Fig). However, the treatment for patients with KFD, AOSD, and SLE was different according to the underlying disease, and patients with KFD had a higher rate of glucocorticoid cessation after 3 months (KFD 66.7%, AOSD 11.1%, SLE 0.0%, p<0.001) and had fewer immunosuppressive agents administered (Table 4).

## Discussion

While KFD is pathologically characterised by a proliferation of activated cytotoxic T lymphocytes and histiocytes with central necrotic areas in lymph nodes, HLH is characterised by hemophagocytosis, engulfing erythrocytes, and leukocytes by histiocyte in the bone marrow or liver. Therefore, both KFD and HLH are associated with abnormal histiocyte activation. In addition, inflammatory cytokines such as tumor necrosis factor (TNF)-α, interleukin (IL)-1, and IL-6 are also implicated in the pathogenesis of both MAS and severe cases of KFD [30, 31]. Therefore, although it is possible that HLH can be accompanied by KFD, the association between KFD and HLH is not well understood. In this study, by using the 2016 EULAR/ACR/PRINTO classification criteria, we found the incidence of MAS, a secondary form of HLH, was 30.8% in our study population of pathologically confirmed KFD. Because we included only febrile hospitalised patients with KFD, our study population might have been skewed towards those having severe laboratory abnormalities in tertiary referral centres; however, the association of MAS in KFD is an interesting finding that requires clinical attention.

KFD patients with MAS experienced longer hospital stays and worse hospitalisation outcomes, including higher rates of intensive care unit stays and in-hospital mortality than in patients without MAS. In addition, the proportion of patients with KFD requiring glucocorticoid treatment was significantly higher in patients with MAS than in patients without MAS. All 5 cases of mortality in our cohort were observed in patients with MAS who were over the age of 50. Compared to patients aged <50 years, those aged 50 years and older had significantly higher mortality in KFD with MAS. These findings suggest that older KFD patients with MAS might be especially associated with poor prognosis.

MAS presents as non-remitting fever, generalised lymphadenopathy, hepatosplenomegaly, pancytopenia, high ferritin, and low fibrinogen levels [32]. Since there are no disease-specific clinical manifestations or laboratory abnormalities of MAS, and many of the symptoms resemble those of KFD itself, the clinician’s suspicion is critical for the diagnosis. In addition, even though hyperferritinemia is a prerequisite in the 2016 criteria for MAS, alternative diagnoses which can be associated with elevated ferritin levels such as sepsis and catastrophic antiphospholipid syndrome should be excluded [33]. In our study, the difference in clinical manifestations between patients with KFD with and without MAS was not significant. On the other hand, in terms of laboratory findings, patients with KFD and MAS had higher CRP, LDH, ferritin, AST, and ALT levels and lower platelet counts, lymphocyte count, and albumin levels than patients with KFD without MAS. Importantly, in our study, a higher proportion of patients with KFD and MAS were treated with steroids than patients without MAS. Monitoring for MAS in patients with KFD could be clinically important, as patients without MAS could be treated with supportive care, while those with MAS might require steroids or additional second-line treatments such as cyclosporine, anti-cytokine therapy, or chemotherapy [34].

Comparison of clinical and laboratory features between patients with MAS-associated KFD, AOSD, and SLE showed that they had different characteristics. Notably, a higher proportion of patients with KFD were able to stop steroids within 3 months compared to patients with SLE or AOSD. Additionally, fewer immunosuppressive agents were administered to patients with MAS and KFD than to patients with MAS and AOSD or SLE. Hence, the clinical course of MAS-associated KFD is milder than MAS with SLE or AOSD, suggesting that the treatment of MAS should be tailored to the underlying diseases.

In our data, 50% (12/24) of KFD patients with MAS received bone marrow evaluation and only 16.7% (2/12) patients were found to have hemophagocytosis. Although this is a small number of patients in a retrospective study, the incidence of hemophagocytosis in our KFD patients were lower than other studies with autoimmune diseases. In a recent review paper of MAS with autoimmune diseases, 42%-100% were positive for bone marrow hemophagocytosis in patients with SLE, Kawasaki disease, and sJIA [35]. In our recent MAS study with AOSD patients, 50% was positive for hemophagocytosis in bone marrow evaluation [26]. It is possible that our KFD patients might have received bone marrow biopsy in the early course which often makes the result negative [21]. It is also possible that macrophage activation in lymph nodes might be enough to drive MAS in KFD. Although in the autoimmune area, bone marrow analysis is not always required for diagnosis of MAS, it is of interest to validate our finding in future studies.

The strength of this study is that, to our knowledge, it is the first study to investigate the incidence and clinical outcomes of MAS in patients with KFD. We only included patients whose diagnoses were confirmed pathologically, further validating our results. Since KFD is prevalent in young Asian women, we have a large population of patients with KFD at our institution, enabling recruitment of a statistically significant number of patients for this study. However, our study also has several limitations. First, it is a retrospective study, and the data were collected by reviewing the hospital’s medical records. Second, there were patients who did not have ferritin, fibrinogen, and triglyceride level results; this might have influenced patient selection and classification resulting in selection bias because clinically severe patients may undergo more blood tests. Therefore, the incidence of MAS in our study might be relevant in patients with KFD and severe clinical or laboratory features. Third, data regarding pro-inflammatory cytokines (TNF-α, interferon-γ, IL-1, IL-6, and soluble CD163) associated with MAS were not obtainable because the following tests were not available in our hospitals [36, 37]. Because these cytokines are known to play an important role in the pathogenesis of MAS [30, 31], assessment of such tests in future studies would provide additional significance. Fourth, because the 2016 classification criteria for MAS have only been validated in patients with sJIA, caution should be made when applying the criteria to non-sJIA diseases. HLH-2004 criteria may be more suitable for non-sJIA diseases. Because a majority of patients had not undergone tests of bone marrow study, natural killer cell activity, and soluble CD25, we could not validate our finding using HLH-2004 criteria.

In conclusion, our findings suggest that the application of the 2016 EULAR/ACR/PRINTO criteria for MAS in hospitalised patients with KFD can be useful in detecting MAS. In addition, although the clinical and laboratory findings were different based on the underlying autoimmune disease, the presence of MAS based on the 2016 EULAR/ACR/PRINTO criteria was associated with adverse patient prognosis. Especially older patients with KFD who presents with features of MAS may be associated with a high mortality rate and may need special attention. Therefore, monitoring patients with KFD according to the 2016 criteria for MAS might aid in predicting patient prognosis and the stratification of high-risk patients.

## Supporting information

S1 Fig Flowchart for patient selection. The flowchart for patient inclusion.
(TIF)

S2 Fig HScore and the probability of having hemophagocytic syndrome among patients with MAS in KFD, AOSD, and SLE. Comparison of HScore (A) and the probability of having hemophagocytic syndrome (B) in patients with MAS and KFD, AOSD, or SLE. HScore; hemophagocytic syndrome score; MAS, macrophage activation syndrome; KFD, Kikuchi-Fujimoto disease; AOSD, adult onset Still’s disease; SLE, systemic lupus erythematosus.
(TIF)

S3 Fig Kaplan-Meier survival curve of 90-day mortality among patients with MAS in KFD, AOSD, and SLE. Comparison of survival probability among patients with MAS in KFD, AOSD, and SLE. MAS, macrophage activation syndrome; KFD, Kikuchi-Fujimoto disease; AOSD, adult onset Still’s disease; SLE, systemic lupus erythematosus.
(TIF)

S1 File The clinical data of study patients. The raw data of this study are shown in the excel file.
(XLSX)

## References

[1] Fujimoto Y. Cervical subacute necrotizing lymphadenitis. A new clinicopathological entity. Intern Med. 1972;20:920–7.

[2] Kikuchi M. Lymphadenitis showing focal reticulum cell hyperplasia with nuclear debris and phagocytes: a clinicopathological study. Acta Haematol Jpn. 1972;35:379–80.

[3] Fiorella ML, Gelardi M, Marzullo A, Sabattini E, Fiorella R. Kikuchi-Fujimoto disease: an uncommon cause of neck swelling. European archives of oto-rhino-laryngology: official journal of the European Federation of Oto-Rhino-Laryngological Societies (EUFOS): affiliated with the German Society for Oto-Rhino-Laryngology—Head and Neck Surgery. 2017;274(3):1761–4. Epub 2016/06/19. 10.1007/s00405-016-4147-6 . 27317565

[4] Hall LD, Eminger LA, Hesterman KS, Heymann WR. Epstein-Barr virus: dermatologic associations and implications: part I. Mucocutaneous manifestations of Epstein-Barr virus and nonmalignant disorders. J Am Acad Dermatol. 2015;72(1):1–19; quiz -20. Epub 2014/12/17. 10.1016/j.jaad.2014.07.034 . 25497917

[5] Mathew LM, Kapila R, Schwartz RA. Kikuchi-Fujimoto disease: a diagnostic dilemma. International journal of dermatology. 2016;55(10):1069–75. Epub 2016/05/22. 10.1111/ijd.13314 . 27207311

[6] Deaver D, Horna P, Cualing H, Sokol L. Pathogenesis, diagnosis, and management of Kikuchi-Fujimoto disease. Cancer Control. 2014;21(4):313–21. Epub 2014/10/14. 10.1177/107327481402100407 . 25310212

[7] Hutchinson CB, Wang E. Kikuchi-Fujimoto disease. Arch Pathol Lab Med. 2010;134(2):289–93. Epub 2010/02/04. . 20121621 10.5858/134.2.289

[8] Bosch X, Guilabert A. Kikuchi-Fujimoto disease. Orphanet journal of rare diseases. 2006;1:18 Epub 2006/05/26. 10.1186/1750-1172-1-18 . 16722618 PMC1481509

[9] Ramirez AL, Johnson J, Murr AH. Kikuchi-Fujimoto’s disease: an easily misdiagnosed clinical entity. Otolaryngology—head and neck surgery: official journal of American Academy of Otolaryngology-Head and Neck Surgery. 2001;125(6):651–3. Epub 2001/12/18. 10.1067/mhn.2001.120431 . 11743471

[10] Nayak HK, Mohanty PK, Mallick S, Bagchi A. Diagnostic dilemma: Kikuchi’s disease or tuberculosis? BMJ case reports. 2013;2013 Epub 2013/02/01. 10.1136/bcr-2012-008026 . 23365168 PMC3604343

[11] Chmait RH, Meimin DL, Koo CH, Huffaker J. Hemophagocytic syndrome in pregnancy. Obstet Gynecol. 2000;95(6 Pt 2):1022–4. Epub 2000/05/16. . 10808012 10.1016/s0029-7844(00)00834-6

[12] Kucukardali Y, Solmazgul E, Kunter E, Oncul O, Yildirim S, Kaplan M. Kikuchi-Fujimoto Disease: analysis of 244 cases. Clin Rheumatol. 2007;26(1):50–4. Epub 2006/03/16. 10.1007/s10067-006-0230-5 . 16538388

[13] Lee HY, Huang YC, Lin TY, Huang JL, Yang CP, Hsueh T, et al Primary Epstein-Barr virus infection associated with Kikuchi’s disease and hemophagocytic lymphohistiocytosis: a case report and review of the literature. J Microbiol Immunol Infect. 2010;43(3):253–7. Epub 2011/02/05. 10.1016/S1684-1182(10)60040-0 . 21291855

[14] Lim GY, Cho B, Chung NG. Hemophagocytic lymphohistiocytosis preceded by Kikuchi disease in children. Pediatr Radiol. 2008;38(7):756–61. Epub 2008/05/23. 10.1007/s00247-008-0894-x . 18496683

[15] Nishiwaki M, Hagiya H, Kamiya T. Kikuchi-Fujimoto Disease Complicated with Reactive Hemophagocytic Lymphohistiocytosis. Acta Med Okayama. 2016;70(5):383–8. Epub 2016/10/26. 10.18926/AMO/54597 . 27777431

[16] Dumas G, Prendki V, Haroche J, Amoura Z, Cacoub P, Galicier L, et al Kikuchi-Fujimoto disease: retrospective study of 91 cases and review of the literature. Medicine (Baltimore). 2014;93(24):372–82. Epub 2014/12/17. 10.1097/md.0000000000000220 . 25500707 PMC4602439

[17] Kim YM, Lee YJ, Nam SO, Park SE, Kim JY, Lee EY. Hemophagocytic syndrome associated with Kikuchi’s disease. J Korean Med Sci. 2003;18(4):592–4. Epub 2003/08/19. 10.3346/jkms.2003.18.4.592 . 12923340 PMC3055072

[18] Marsili M, Nozzi M, Onofrillo D, Sieni E, Chiarelli F, Breda L. Kikuchi disease, macrophage activation syndrome, and systemic juvenile arthritis: a new case associated with a mutation in the perforin gene. Scandinavian journal of rheumatology. 2015;44(5):429–30. Epub 2015/05/15. 10.3109/03009742.2015.1033009 . 25974073

[19] Hayden A, Park S, Giustini D, Lee AY, Chen LY. Hemophagocytic syndromes (HPSs) including hemophagocytic lymphohistiocytosis (HLH) in adults: A systematic scoping review. Blood reviews. 2016;30(6):411–20. Epub 2016/05/31. 10.1016/j.blre.2016.05.001 . 27238576

[20] Grom AA, Mellins ED. Macrophage activation syndrome: advances towards understanding pathogenesis. Curr Opin Rheumatol. 2010;22(5):561–6. Epub 2010/06/03. 10.1097/01.bor.0000381996.69261.71 . 20517154 PMC4443835

[21] Grom AA, Horne A, De Benedetti F. Macrophage activation syndrome in the era of biologic therapy. Nat Rev Rheumatol. 2016;12(5):259–68. Epub 2016/03/25. 10.1038/nrrheum.2015.179 . 27009539 PMC5851441

[22] Song Y, Wang Y, Wang Z. Requirement for etoposide in the initial treatment of Epstein-Barr virus-associated haemophagocytic lymphohistiocytosis. Br J Haematol. 2019 Epub 2019/05/23. 10.1111/bjh.15988 . 31115044

[23] Ravelli A, Minoia F, Davi S, Horne A, Bovis F, Pistorio A, et al 2016 Classification Criteria for Macrophage Activation Syndrome Complicating Systemic Juvenile Idiopathic Arthritis: A European League Against Rheumatism/American College of Rheumatology/Paediatric Rheumatology International Trials Organisation Collaborative Initiative. Ann Rheum Dis. 2016;75(3):481–9. Epub 2016/02/13. 10.1136/annrheumdis-2015-208982 . 26865703

[24] Ravelli A, Minoia F, Davi S, Horne A, Bovis F, Pistorio A, et al 2016 Classification Criteria for Macrophage Activation Syndrome Complicating Systemic Juvenile Idiopathic Arthritis: A European League Against Rheumatism/American College of Rheumatology/Paediatric Rheumatology International Trials Organisation Collaborative Initiative. Arthritis Rheumatol. 2016;68(3):566–76. Epub 2015/09/01. 10.1002/art.39332 . 26314788

[25] Ahn SS, Yoo BW, Jung SM, Lee SW, Park YB, Song JJ. In-hospital mortality in febrile lupus patients based on 2016 EULAR/ACR/PRINTO classification criteria for macrophage activation syndrome. Semin Arthritis Rheum. 2017 Epub 2017/03/08. 10.1016/j.semarthrit.2017.02.002 . 28268026

[26] Ahn SS, Yoo BW, Jung SM, Lee SW, Park YB, Song JJ. Application of the 2016 EULAR/ACR/PRINTO Classification Criteria for Macrophage Activation Syndrome in Patients with Adult-onset Still Disease. J Rheumatol. 2017 Epub 2017/04/17. 10.3899/jrheum.161286 . 28412707

[27] Fardet L, Galicier L, Lambotte O, Marzac C, Aumont C, Chahwan D, et al Development and validation of the HScore, a score for the diagnosis of reactive hemophagocytic syndrome. Arthritis Rheumatol. 2014;66(9):2613–20. Epub 2014/05/02. 10.1002/art.38690 . 24782338

[28] Batu ED, Erden A, Seyhoglu E, Kilic L, Buyukasik Y, Karadag O, et al Assessment of the HScore for reactive haemophagocytic syndrome in patients with rheumatic diseases. Scandinavian journal of rheumatology. 2017;46(1):44–8. Epub 2016/07/01. 10.3109/03009742.2016.1167951 . 27359073

[29] Jordan MB, Allen CE, Weitzman S, Filipovich AH, McClain KL. How I treat hemophagocytic lymphohistiocytosis. Blood. 2011;118(15):4041–52. Epub 2011/08/11. 10.1182/blood-2011-03-278127 . 21828139 PMC3204727

[30] Schulert GS, Grom AA. Macrophage activation syndrome and cytokine-directed therapies. Best practice & research Clinical rheumatology. 2014;28(2):277–92. Epub 2014/06/30. 10.1016/j.berh.2014.03.002 . 24974063 PMC4074772

[31] Barbat B, Jhaj R, Khurram D. Fatality in Kikuchi-Fujimoto disease: A rare phenomenon. World journal of clinical cases. 2017;5(2):35–9. Epub 2017/03/04. 10.12998/wjcc.v5.i2.35 . 28255545 PMC5314258

[32] Behrens EM. Macrophage activation syndrome in rheumatic disease: what is the role of the antigen presenting cell? Autoimmunity reviews. 2008;7(4):305–8. Epub 2008/02/26. 10.1016/j.autrev.2007.11.025 . 18295734

[33] Agmon-Levin N, Rosario C, Katz BS, Zandman-Goddard G, Meroni P, Cervera R, et al Ferritin in the antiphospholipid syndrome and its catastrophic variant (cAPS). Lupus. 2013;22(13):1327–35. Epub 2013/09/17. 10.1177/0961203313504633 . 24036580

[34] Shakoory B, Carcillo JA, Chatham WW, Amdur RL, Zhao H, Dinarello CA, et al Interleukin-1 Receptor Blockade Is Associated With Reduced Mortality in Sepsis Patients With Features of Macrophage Activation Syndrome: Reanalysis of a Prior Phase III Trial. Critical care medicine. 2016;44(2):275–81. Epub 2015/11/20. 10.1097/CCM.0000000000001402 . 26584195 PMC5378312

[35] Lerkvaleekul B, Vilaiyuk S. Macrophage activation syndrome: early diagnosis is key. Open access rheumatology: research and reviews. 2018;10:117–28. Epub 2018/09/15. 10.2147/oarrr.S151013 . 30214327 PMC6124446

[36] Colafrancesco S, Priori R, Alessandri C, Astorri E, Perricone C, Blank M, et al sCD163 in AOSD: a biomarker for macrophage activation related to hyperferritinemia. Immunologic research. 2014;60(2–3):177–83. Epub 2014/11/13. 10.1007/s12026-014-8563-7 . 25388964

[37] Crayne CB, Albeituni S, Nichols KE, Cron RQ. The Immunology of Macrophage Activation Syndrome. Frontiers in immunology. 2019;10:119 Epub 2019/02/19. 10.3389/fimmu.2019.00119 . 30774631 PMC6367262

