# Supplementary figures and images for: Evaluation of macrophage activation syndrome in hospitalised patients with Kikuchi-Fujimoto disease based on the 2016 EULAR/ACR/PRINTO classification criteria
Source: PLoS One. 2019 Jul 18;14(7):e0219970. doi: 10.1371/journal.pone.0219970 (PMC6638985; doi:10.1371/journal.pone.0219970)

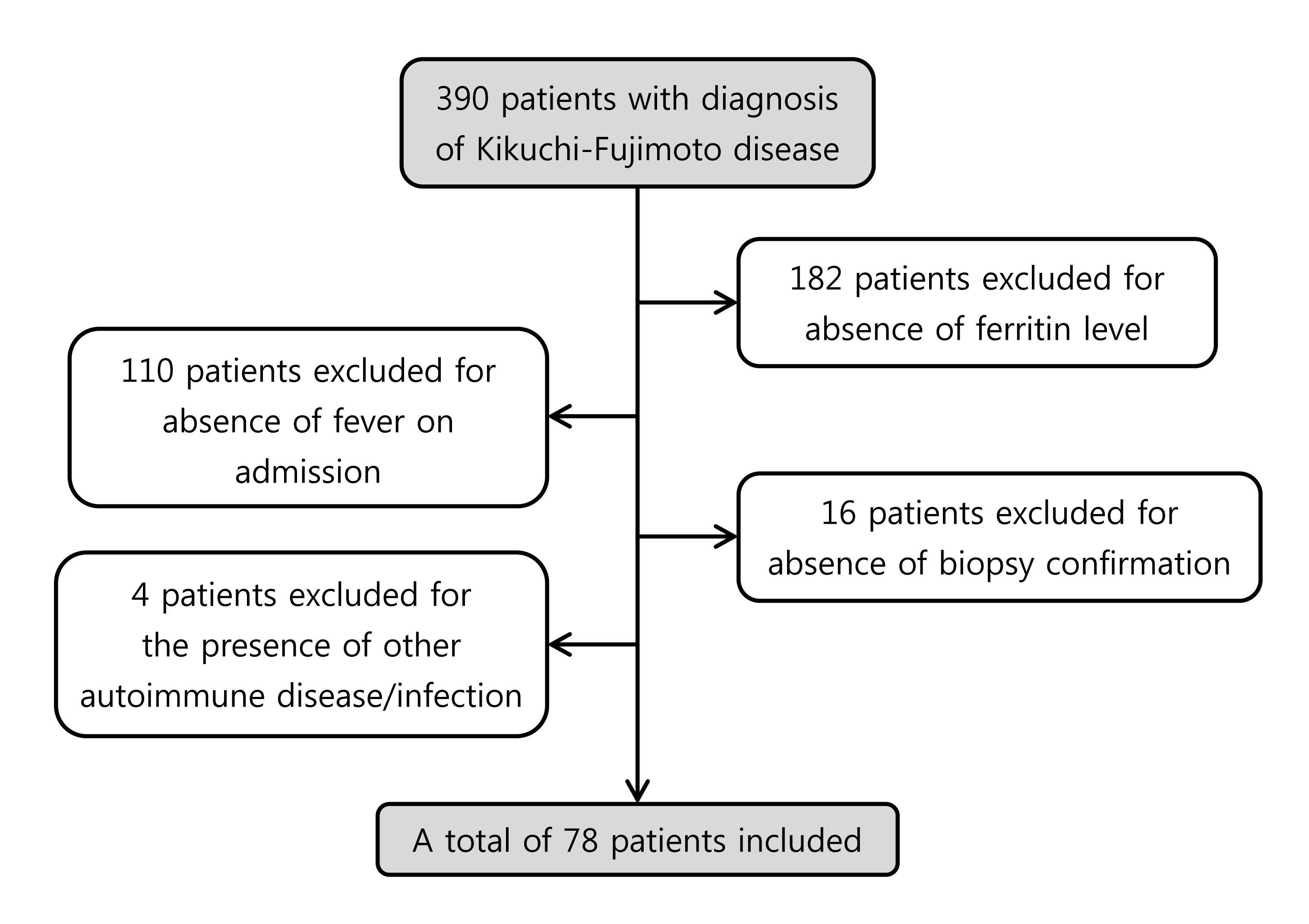

Supplement: S1 Fig — The flowchart for patient inclusion. (TIF) [file pone.0219970.s001.tif]

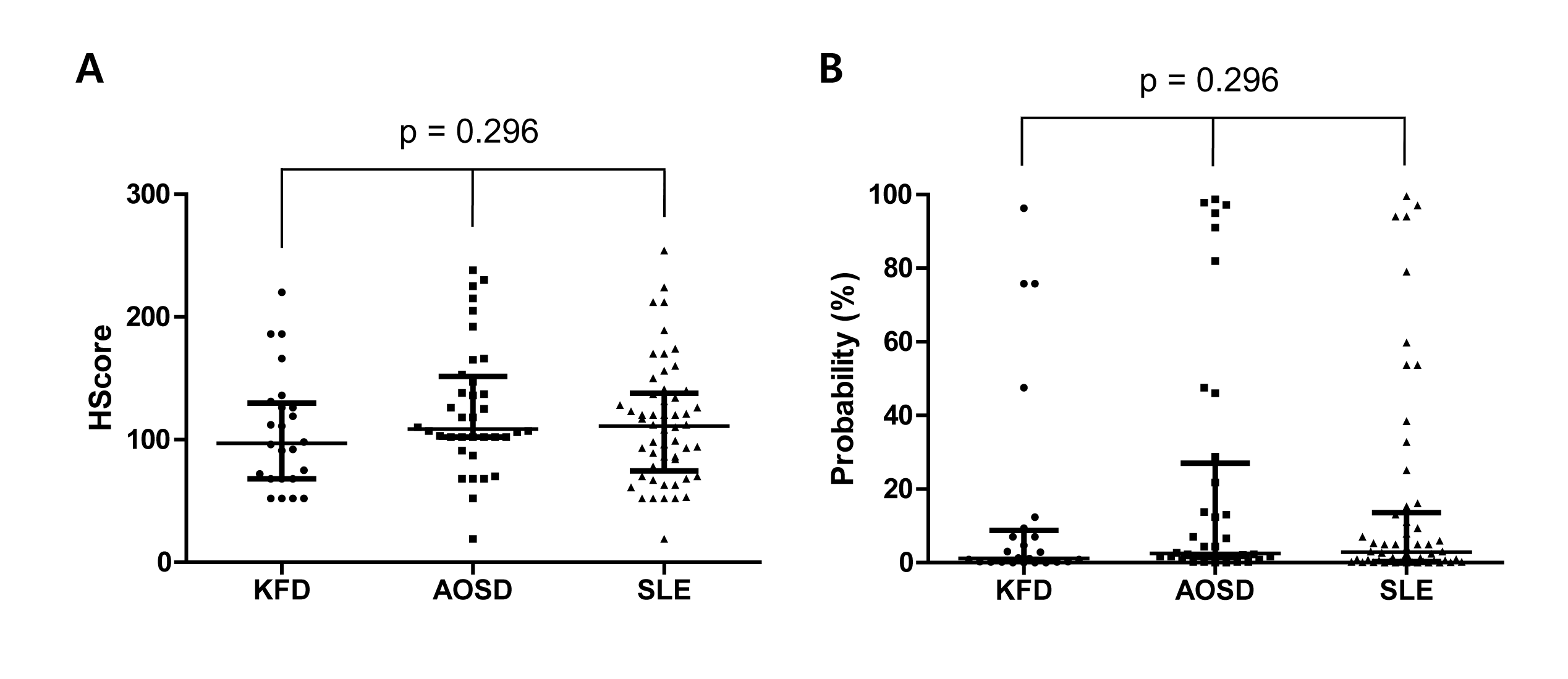

Supplement: S2 Fig — Comparison of HScore (A) and the probability of having hemophagocytic syndrome (B) in patients with MAS and KFD, AOSD, or SLE. HScore; hemophagocytic syndrome score; MAS, macrophage activation syndrome; KFD, Kikuchi-Fujimoto disease; AOSD, adult onset Still’s disease; SLE, systemic lupus erythematosus. (TIF) [file pone.0219970.s002.tif]

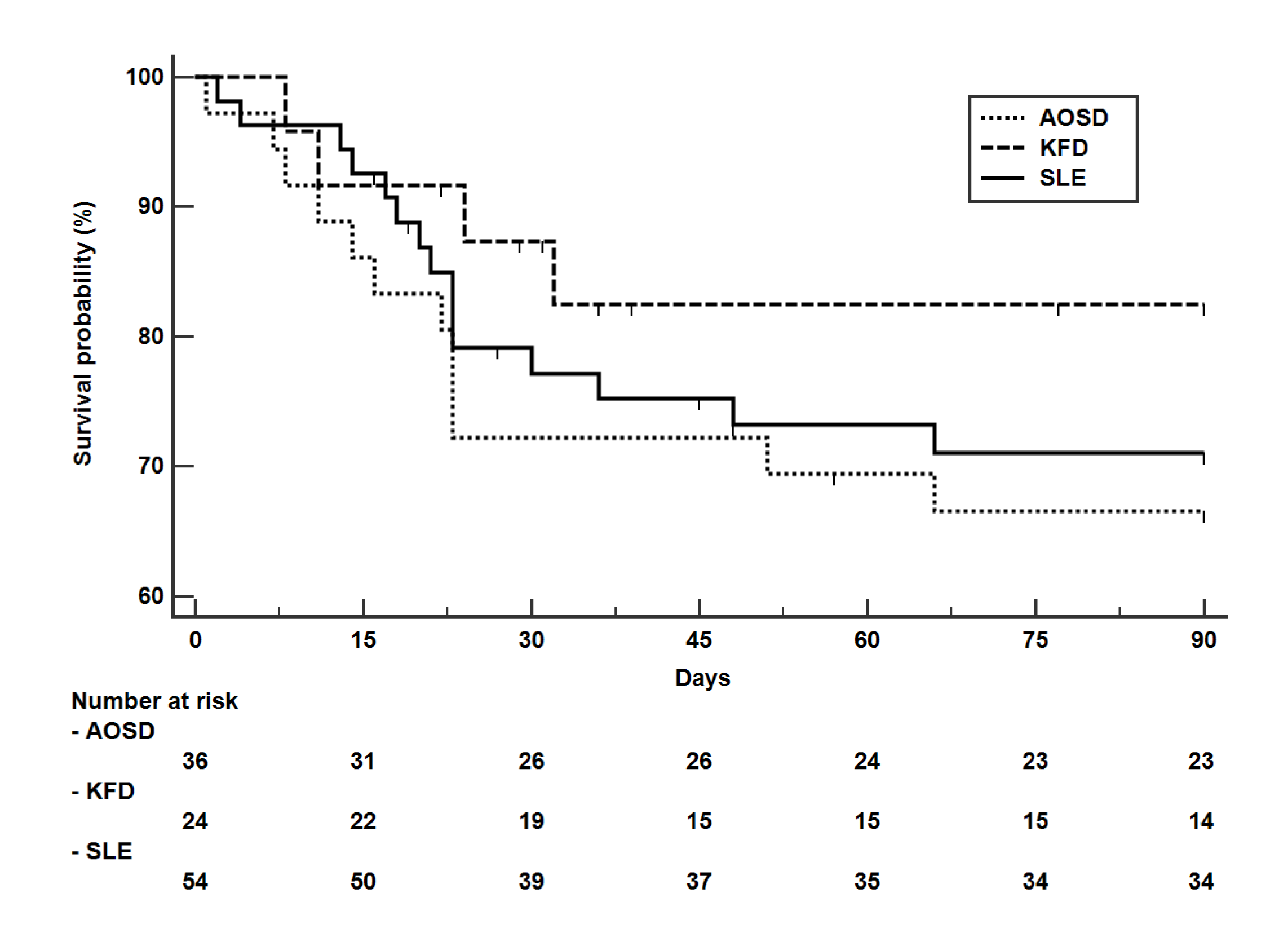

Supplement: S3 Fig — Comparison of survival probability among patients with MAS in KFD, AOSD, and SLE. MAS, macrophage activation syndrome; KFD, Kikuchi-Fujimoto disease; AOSD, adult onset Still’s disease; SLE, systemic lupus erythematosus. (TIF) [file pone.0219970.s003.tif]
